# Individual variation and repeatability of methane production from dairy cows estimated by the CO_2_ method in automatic milking system

**DOI:** 10.1017/S1751731115000646

**Published:** 2015-05-08

**Authors:** M. N. Haque, C. Cornou, J. Madsen

**Affiliations:** Department of Large Animal Sciences, Faculty of Health and Medical Sciences, University of Copenhagen, Groennegaardsvej 2, DK-1870 Frederiksberg C, Denmark

**Keywords:** breath, diurnal variation, methane, phenotypic correlation, dairy cows

## Abstract

The objectives of this study were to investigate the individual variation, repeatability and correlation of methane (CH_4_) production from dairy cows measured during 2 different years. A total of 21 dairy cows with an average BW of 619±14.2 kg and average milk production of 29.1±6.5 kg/day (mean±s.d.) were used in the 1^st^ year. During the 2^nd^ year, the same cows were used with an average BW of 640±8.0 kg and average milk production of 33.4±6.0 kg/day (mean±s.d.). The cows were housed in a loose housing system fitted with an automatic milking system (AMS). A total mixed ration was fed to the cows *ad libitum* in both years. In addition, they were offered concentrate in the AMS based on their daily milk yield. The CH_4_ and CO_2_ production levels of the cows were analysed using a Gasmet DX-4030. The estimated dry matter intake (EDMI) was 19.8±0.96 and 23.1±0.78 (mean±s.d.), and the energy-corrected milk (ECM) production was 30.8±8.03 and 33.7±5.25 kg/day (mean±s.d.) during the 1^st^ and 2^nd^ year, respectively. The EDMI and ECM had a significant influence (*P*<0.001) on the CH_4_ (l/day) yield during both years. The daily CH_4_ (l/day) production was significantly higher (*P*<0.05) during the 2^nd^ year compared with the 1^st^ year. The EDMI (described by the ECM) appeared to be the key factor in the variation of CH_4_ release. A correlation (*r*=0.54) of CH_4_ production was observed between the years. The CH_4_ (l/day) production was strongly correlated (*r*=0.70) between the 2 years with an adjusted ECM production (30 kg/day). The diurnal variation of CH_4_ (l/h) production showed significantly lower (*P*<0.05) emission during the night (0000 to 0800 h). The between-cows variation of CH_4_ (l/day, l/kg EDMI and l/kg ECM) was lower compared with the within-cow variation for the 1^st^ and 2^nd^ years. The repeatability of CH_4_ production (l/day) was 0.51 between 2 years. In conclusion, a higher EDMI (kg/day) followed by a higher ECM (kg/day) showed a higher CH_4_ production (l/day) in the 2^nd^ year. The variations of CH_4_ (l/day) among the cows were lower than the within-cow variations. The CH_4_ (l/day) production was highly repeatable and, with an adjusted ECM production, was correlated between the years.

## Implications

Daily methane (CH_4_) production is different between cows. CH_4_ production mainly depends on the feed intake, which is related to the milk production. The variation of CH_4_ production remained even after the standardization of the feed intake and milk yield. This animal variation can most likely be used to select cows with low CH_4_ production as a long-term mitigation approach. For the selection of the correct low CH_4_ emitting cows, it is important that the measured low emission can be repeated. This experiment shows that the ranking of the cows can be repeated over different years.

## Introduction

The livestock sector represents a significant source of greenhouse gas (GHG) emissions worldwide, generating carbon dioxide (CO_2_), methane (CH_4_) and nitrous oxide throughout the production process. This sector is often the focus of study because of its large impact on the environment. A recent report by Gerber *et al.* (2013) described that the majority of CH_4_ emissions occurred from the livestock sector as a result of enteric fermentation and feed production. In the livestock sector, cattle are the highest contributors of GHG emissions; the GHG emissions from cattle account for 65% of the GHG emissions from the livestock sector (4.6 Gt CO_2_ eq). Of the total emissions, cattle emit the most enteric CH_4_, that is, ~77%, followed by the other domesticated species (Gerber *et al.*, 2013). Another consideration in addition to environmental pollution is that between 2% and 12% of the ingested gross energy is lost through CH_4_ emission (Johnson and Johnson, 1995); this loss of energy could potentially be used by the animals. The CH_4_ emissions from the animals vary according to the level of feed intake, type of carbohydrate, type of feed processing, addition of lipids, alteration of rumenal microflora (Johnson and Johnson, 1995) and measurement techniques (Vlaming *et al.*, 2008). In addition, it can also vary as a result of the genetic variation of the animals (Pinares-Patiño *et al.*, 2013). One of the earlier studies using a standard respiration chamber reported a CV of 7% for within-animal variation for CH_4_ production and of 7% to 8% for between-animal variation (Blaxter and Clapperton, 1965). More recently, several authors reported a CV of 4.3% for within-animal variation and 17.8% for between-animal variation using open-circuit calorimetry (Grainger *et al.*, 2007). Using the SF_6_ technique, Vlaming *et al.* (2008) mentioned a wider range of variation in CH_4_ emissions for two different diets (6.91% to 10.09% for within cow and 6.23% to 27.79% for between cow). Moreover, under grazing conditions, Lassey *et al.* (1997), Boadi *et al.* (2002) and McNaughton *et al.* (2005) reported between-animal variations of 11.5%, 15.5% and 25% CV, respectively, using the SF_6_ technique. In a comparative study using two different techniques, Grainger *et al.* (2007) mentioned a higher within-cow variation (CV=19.6%) for SF_6_ techniques compared with the chamber technique (CV=17.8%). To date, most studies have estimated the animal variation in CH_4_ production, either by using the traditional chamber technique or SF_6_ techniques, where handling and confinement of the animals is required. A drawback of these methods is that they might have an influence on the normal metabolism of the animals. In this study, we assume that the animal should be free from any influential factors to understand individual variability in CH_4_ production. We hypothesize that CH_4_ production resulting from animal variation would be lower if the measurements are taken from their natural environment. In the dairy industry, automatic milking systems (AMS) reduce human involvement and interactions with cows, thus allowing the cows to have free movement. Therefore, under this condition, normal feeding and milking behaviour as well as rumen metabolism and gas production can be expected. The ‘CO_2_ method’, a newly developed technique for CH_4_ estimation, was used in this study. This method is non-invasive and measures the CH_4_ production from cows by keeping them in their natural environment. The objectives of this study were (i) to investigate individual variation and CH_4_ production repeatability measured in an AMS and (ii) to investigate the correlation of CH_4_ production of individual cows during 2 different years.

## Material and methods

### Animals, experimental design and feeding

A total of 21 dairy cows with an average BW of 619±14.2kg and average milk production of 29.1±6.5kg/day (mean±s.d.) were used in the 1^st^ year. Among the total number of cows, 14 were primiparous and seven were multiparous in the 1^st^ year. The cows were in the same lactation stage, with an approximate calving interval of 12 months. During the 2^nd^ year, the same cows were used, with an average BW of 640±8.0 kg and average milk production of 33.4±6.0 kg/day (mean±s.d.). The cows were housed in a loose housing system that had adequate ventilation and was fitted with an AMS. The study was conducted without interfering with the feeding and management planned by the farm. During both years, the measurements were taken from the same cows in the same AMS. The experimental period was 7 days in the 2^nd^ week of May each year. The cows were offered a total mixed ration (TMR) *ad libitum* (Table 1) in both years. In addition to the TMR, they were offered concentrate in the AMS based on their daily average milk production. The TMR was allocated in the morning at ~0700 h, and at ~1500 h, the remaining feed residuals were mixed and moved closer to the cow. A total of 57 cows were milked in the AMS; of these 57, 23 cows were common in both years. Among the common cows, two cows showed abnormal milking behaviour. One cow had just calved and only visited the AMS for 3 of the 7 days of measurements. The other cow visited the AMS once per day and was treated for lameness. These two cows were therefore excluded from the analysis; thus, 21 cows were studied.

**Table 1:** Feed allocation and nutrient composition of diet over the 2 years

Ingredients	1^st^ year (DM, kg/day)	2^nd^ year (DM, kg/day)
Total mixed ration		
Rapeseed cake	1.6	1.3
Soybean decorticated	1.4	1.0
Clover grass silage	3.4	2.9
Maize silage	9.0	10.7
Ryegrass straw	0.6	1.4
Urea	–	0.1
Beet pulp	–	0.9
Vitamin mineral premix	0.2	0.2
Concentrate supplied in AMS		
Concentrate	4.0	4.2
Nutrient intake^1^		
Energy (MJ/kg DM)^2^	7.6	6.3
MEI (MJ/cow per day)	153.0	146.0
AAT (g/MJ)	13.0	16.0
PBV (g/kg DM)	5.0	8.0
Fatty acid (g/kg DM)	35.0	28.0
NDF (g/kg DM)	–	342.1
Starch (g/kg DM)	212.3	199.1
Calcium total (g/day)	147.0	143.0
Total phosphorus (g/day)	84.6	78.3
Magnesium total (g/day)	58.2	56.0

AMS=automatic milking system; DM=dry matter; MEI=metabolizable energy intake; AAT=amino acids absorbed in the small intestine; PBV=protein balance in the rumen.

1 Nutrient and energy values were calculated using the Danish feed stuffs table (Møller *et al.*, 2000).

2 Net energy for feed utilization (Nørgaard *et al.*, 2011).

### Gas measurement

The CH_4_ and CO_2_ production levels of the cows was analysed using a continuous gas analyser, the ‘Gasmet DX-4030’ (Gasmet Technologies Oy, Helsinki, Finland), based on Fourier transformed IR. The inlet filter of the Gasmet was fitted on the feeding pen of the AMS to obtain concentrated breath samples from individual cows. The breath samples pass through the inlet filter and then through the Gasmet to determine the concentration of CH_4_ and CO_2_. The measurements were performed every 15 s over 24 h for 7 consecutive days during milking in the AMS. Each individual cow visited the AMS at least two times per day (ranging from 1 to 4, average 2.54). Before the first measurement, the Gasmet was calibrated with standard gases to check the accuracy of the measurements. The Gasmet was disconnected for 10 min randomly during each measurement day to obtain the barn concentration of CH_4_ and CO_2_. The average of this concentration was used as a correction factor for the entire experimental period to obtain the actual breath concentration of CH_4_ and CO_2_. The measurements were remotely monitored via the internet using TeamViewer.

### Calculations

Identification numbers and the entrance and exit times of each individual cow were recorded in a computer connected to the AMS. These data were matched with the breath analysis data from the Gasmet. All of the calculations regarding the CH_4_ estimation were performed according to the CO_2_ method (Madsen *et al.*, 2010). The protocol of the method is described in the following three steps.

*Step I: Calculation of the CH*_*4*_ : *CO*_*2*_ *ratio.* The CO_2_ method uses the measured CH_4_ : CO_2_ ratio from the breath sample analysis of the individual cows. The average barn concentrations of CH_4_ (23.2 and 25.8 ppm) and CO_2_ (495.8 and 625.5 ppm) were obtained during measurements in the 1^st^ and 2^nd^ year, respectively. These concentrations were subtracted from the exhaled concentrations to get the corrected CH_4_ and CO_2_ (ppm) of the individual cows. The data that were below 400 ppm for the corrected CO_2_ were removed to avoid the influence of samples that contained a very low concentration of CH_4_ and CO_2_ (ppm). The ratio between CH_4_ and CO_2_ (CH_4_ : CO_2_) was thereafter calculated.

*Step II: Calculation of the total CO*_*2*_ *production per day.* To calculate the total CO_2_ production from the individual cows, it is necessary to first calculate the total heat production (HP). The HP of the cows was calculated according to equation (1) using the cows’ body mass, milk production and number of days pregnant as described by CIGR (2002). Thereafter, the total CO_2_ production per day was calculated according to Pedersen *et al.* (2008), as shown in equation (2).

*Step III: CH*_*4*_ *estimation.* The amount of CH_4_ was calculated according to equation (3). This *uses* the CH_4_ : CO_2_ ratio (described in step I) multiplied by the total CO_2_ production per day (described in step II) and results in the amount of CH_4_ produced.

The concentrate intake in the AMS was measured individually on a daily basis while the TMR intake was considered to be a herd average. The total estimated dry matter intake (EDMI, kg/day) was calculated by adding the individually recorded concentrate dry matter intake (DMI) (kg/day) to the corrected TMR dry matter intake (kg/day) using equation (4) according to Kristensen and Ingvartsen (2003). In this case, a supplementation rate of 0.5 was considered for the concentrate intake. The actual energy-corrected milk (ECM, kg/day) was calculated using equation (5), according to Sjaunja *et al.* (1991). Standardized CH_4_ production and CH_4_ : CO_2_ ratios were calculated at the adjusted 30 (kg/day) ECM level according to equations (6) and (7).(1)
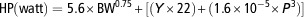

(2)
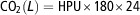

(3)
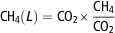

(4)
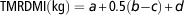

(5)
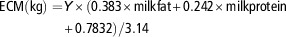

(6)
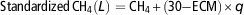

(7)
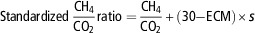
where *a* is the average TMR intake; *b* the average concentrate intake; *c* the concentrate intake of the individual cows during the experimental periods; *d* the correction factor for the lactation number; *d*=−1.61 was used for first lactation and *d*=0.39 was used for the second and subsequent lactations; HP the heat production of the animals; BW^0.75^ the metabolic BW of the animals; *Y* the milk yield of the cows; *P* the number of days the cows were pregnant; *s* the slope of the regression of CH_4_ : CO_2_ ratio as a function of ECM in each year separately; *q* the slope of the regression of CH_4_ as a function of ECM in each year separately; HPU=heat producing unit 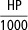 ; 180=*L* of CO_2_/HPU per h; ECM the energy-corrected milk.

### Statistical analyses

Data were analysed with linear mixed models using the lmer function fitted by the restricted maximum likelihood from the package ‘lme4’ (Bates and Sarkar, 2009) using R software (R Development Core Team, 2013). An extension package ‘lmerTest’ was used to obtain the *P* value directly from the lmer function (Kuznetsova *et al.*, 2012). Individual 24-h mean emissions were considered for the interpretation of the results. The analyses focused on making inferences on the individual variation and repeatability of CH_4_ production (l/day, l/kg EDMI and l/kg ECM). The models were fitted on the yearly data subset. The BW, EDMI, ECM, parity and days of pregnancy were included as fixed effects in the primary model that was fitted with the maximum likelihood method. Cows and the number of visits to the AMS were included as random effects. The final model (equation (6)) was confirmed by the stepwise elimination of non-significant variables. The significance of the fixed effects was assessed by *F*-ratio tests, and the significance of the random effects was assessed by likelihood-ratio tests. Model validations were performed with ANOVA based on the Akaike Information Criterion. The model residuals were checked for normality by visual inspection of qqplots. The final model is:
(8)
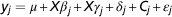

where *y*_*j*_ is the response variable *y*=(CH_4_ (l/day), CH_4_ (l/kg EDMI), CH_4_ (l/kg ECM) and CH_4_ : CO_2_ ratio) of cow *j* and *µ* the overall mean. The fixed effects are the *Xβ*_*j*_=EDMI (kg/day) of cow *j*; *Yγ*_*j*_=ECM (kg/day) of cow *j*; *δ*_*j*_=parity of cow *j*; *C*_*j*_ *=*random effect of cow *j* and *ε*_*j*_ are the residual errors. Model estimates were extracted using the glht function from the ‘multcomp’ package (Hothorn *et al.*, 2008). The CVs of CH_4_ production between cows (CV_bc_) and within cow (CV_wc_) were calculated from the variance components of the model (equation (8)) using equations (9) and (10). The variance components were defined as the ratio of the individual random effect ( 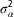 ) and the variance of the random error ( 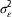 ) to the estimated mean 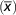.(9)
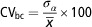

(10)
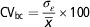
The variance components from the same model (equation (8)) were used to obtain the repeatability (*R*) within a given year, calculated as the proportion of between-animal variation with respect to the total variance as:(11)
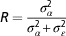
The differences of CH_4_ production between the 2 years were assessed by the following model:(12)
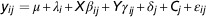
where *λ*_*i*_ is the year of measurement with *i*=1 : 2 years; *Xβ*_*ij*_ the EDMI (kg/day) of year *i* and cow *j*; *Yγ*_*i*_ the ECM (kg/day) of year *i* and cow *j*; *δ*_*j*_ the parity of cow *j*; *C*_*j*_ the random effect of cow and *ε*_*ij*_ are the residual errors. The between-year repeatability (*R*_2_) of CH_4_ production was calculated using the variance components of the model fitted with EDMI (kg/day), ECM (kg/day) and parity as fixed effects and the year of the measurements as the random effect.

Yearly data subsets of the daily mean emissions during milking were considered for the visualization of the diurnal variation of CH_4_ production following the model (equation (13)).(13)
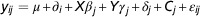
where *μ* is the overall mean; 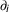 the hours of measurements in a day with *i*=1:24 h; *Xβ*_*j*_ the EDMI (kg/day) of cow *j*; *Y**γ***_*j*_ the ECM (kg/day) of cow *j*; *δ*_*j*_ the parity of cow *j*; *C*_*j*_ the random effect of cow *j* and *ε*_*ij*_ are the residual errors.

## Results

### Feed intake, milk and CH_4_ production in 2 years

**Figure 1:**
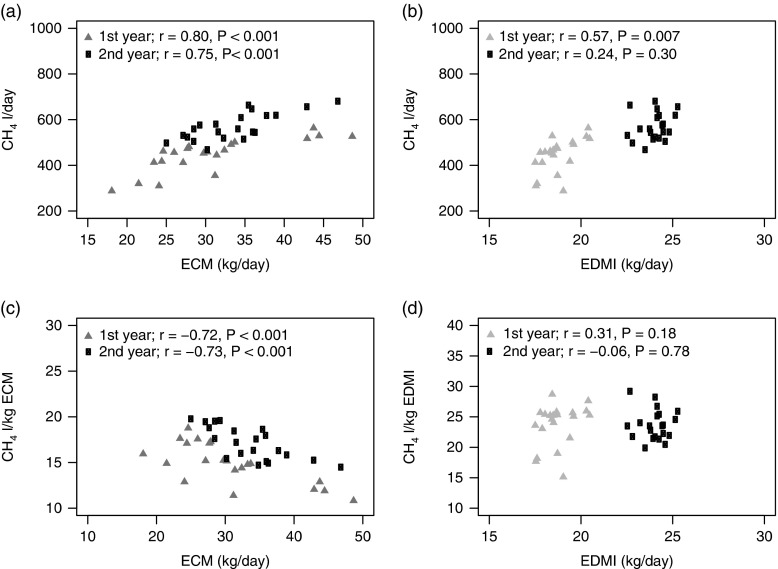
Regression analysis of the CH_4_ production, ECM and EDMI of individual cows over the 2 years. The figure on the left-hand side (a and c) displays CH_4_ (l/day and l/kg ECM) according to ECM (kg/day); whereas the right-hand side (b and d) plots CH_4_ (l/day and l/kg EDMI) according to EDMI (kg/day). The *r*=Pearson’s correlation coefficient and *P* values indicate the significance of the correlation test. ECM=energy-corrected milk; EDMI=estimated dry matter intake.

BW (kg), milk production (kg/day), ECM (kg/day) and EDMI (kg/day) were higher during the 2^nd^ year compared with the 1^st^ year (Table 2). The CH_4_ production (l/day) was positively correlated with the ECM (kg/day) in both years (Figure 1a). A correlation was observed between CH_4_ production (l/day) and EDMI (kg/day) during the 1^st^ year (Figure 1b). However, CH_4_ production (l/day) and EDMI (kg/day) were not correlated during the 2^nd^ year (Figure 1b). The CH_4_ production (l/kg ECM) revealed a negative correlation with the ECM (kg/day) in both years (Figure 1c). However, no correlation was found when the amount of CH_4_ (l/kg EDMI) was plotted against the EDMI (kg/day) (Figure 1d).

**Table 2:** BW, milk production and feed intake of the cows during the 2 years of measurement

Parameters	1^st^ year	2^nd^ year
BW (kg)	619.9±14.2	640.0±8.0
Milk yield (kg/day)	29.1±6.5	33.4±6.0
ECM (kg/day)	30.8±8.0	33.7±5.3
TMRI (DM, kg/day)	15.8±0.5	18.9±0.5
CI (DM, kg/day)	4.0±1.0	4.2±1.6
EDMI (kg/day)	19.8±1.0	23.1±0.8

ECM=energy-corrected milk; TMRI=total mixed ration intake; DM=dry matter; CI=concentrate intake; EDMI=estimated dry matter intake.

Values indicated arithmetic means and standard deviations (mean±s.d.).

### Variation of CH_4_ production in 2 years

CH_4_ production, along with its variability and repeatability, were obtained from the fitted model (equation (6)) using the yearly data subsets (Table 3). The daily production of CH_4_ (l/day and l/kg ECM) was significantly lower (*P*<0.05) in the 1^st^ year compared with the 2^nd^ year. However, CH_4_ (l/kg EDMI) was similar in both years. The between-cow variation of CH_4_ emissions (l/day, l/kg EDMI and l/kg ECM) was lower (CV_bc_=8.8% to 9.1%) than the within-cow variation (CV_wc_=15.7 to 16.4) during the 1^st^ year. The range of the variation during the 2^nd^ year was narrower (CV_bc_=5.9 to 6.1 and CV_wc_=8.6 to 9.1) compared with that of the 1^st^ year. Similarly, variations of the CH_4_ : CO_2_ ratios were lower during the 2^nd^ year (CV_bc_=6.2 and CV_wc_=8.8) compared with the variations during the 1^st^ year (CV_bc_=8.4 and CV_wc_=15.9).

**Table 3:** Variation and repeatability of the CH_4_ production of the cows over 2 years

	1^st^ year				2^nd^ year				
Parameters	Estimates^1^	CV_bc_ (%)	CV_wc_ (%)	*R*	Estimates^1^	CV_bc_ (%)	CV_wc_ (%)	*R*	*R*_2_
CH_4_ (l/day)	445.50	8.80	15.67	0.36	569.88	5.88	8.60	0.41	0.51
CH_4_ (l/kg EDMI)	23.73	9.12	15.70	0.37	23.70	6.01	8.57	0.41	0.49
CH_4_ (l/kg ECM)	14.86	8.96	16.36	0.35	17.10	6.10	9.05	0.40	0.45
Ratio	0.08	8.38	15.94	0.34	0.09	6.22	8.78	0.41	0.45

CV_bc_=coefficient of variation for between-cow variation; CV_wc_=coefficient of variation for within-cow variation; *R*=repeatability within a year; *R*_2_=repeatability between the 2 years; EDMI=estimated dry matter intake; ECM=energy-corrected milk; Ratio=CH_4_ and CO_2_ ratio.

1 Estimates from the model.

### Correlation of CH_4_ production between 2 years

**Figure 2:**
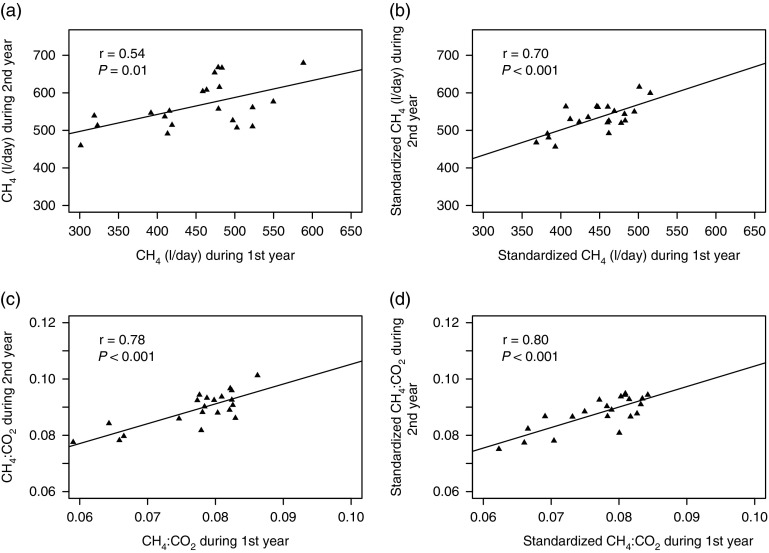
Methane production and CH_4_ : CO_2_ ratios of the individual cows over the 2 years. The left-hand side (a and c) shows the mean CH_4_ (l/day) and CH_4_ : CO_2_ ratios at the actual ECM production; whereas the right-hand side (b and d) visualizes the standardized CH_4_ (l/day) and CH_4_ : CO_2_ ratios calculated at 30 (kg/day) ECM production. The *r*=Pearson’s correlation coefficient and *P* values indicate the significance of the correlation test. ECM=energy-corrected milk.

The individual mean emissions over 7 days were used to establish the correlation of CH_4_ emissions between years. A correlation (*r*=0.54) was observed in the CH_4_ emission between the 2 years in the actual ECM (kg/day) production (Figure 2a). This correlation was increased (*r*=0.70) when it was calculated with an adjusted ECM production (30 kg/day) (Figure 2b). The yearly difference of CH_4_ (l/day) in the actual ECM (kg/day) production was more (*P*=0.008) compared with the difference in the adjusted ECM production (*P*=0.01). However, the CH_4_ : CO_2_ ratio was significantly (*P*<0.001) different between years in both the actual and adjusted ECM (kg/day) production. The correlation of the CH_4_ : CO_2_ ratio between years was slightly increased (*r*=0.80) in the adjusted ECM compared with the value (*r*=0.78) of the actual ECM production (Figure 2c and d).

### Repeatability of CH_4_ production

The within-year repeatability (*R*) of CH_4_ production (l/day, l/kg EDMI and l/kg ECM) was lower (0.35 to 0.37) during the 1^st^ year than in the 2^nd^ year (0.40 to 0.41). The observed repeatability between years (*R*_2_) was 0.51 to 0.45 for the same parameters (Table 3). Likewise, the CH_4_ : CO_2_ ratio was more repeatable in the 2^nd^ year (0.41) compared with the observed *R* during the 1^st^ year (0.34), whereas the resultant *R*_2_ of the CH_4_ : CO_2_ ratio was 0.45 (Table 3).

### Diurnal variation of CH_4_ production

**Figure 3:**
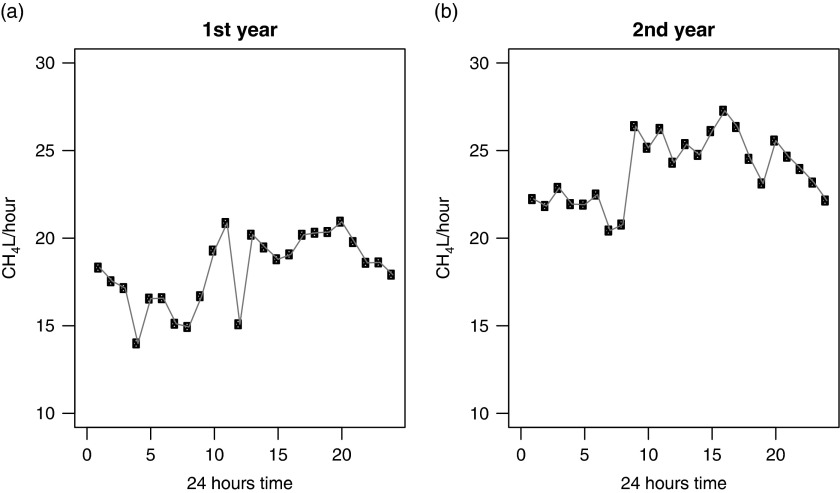
Diurnal variation of CH_4_ release (l/h) over the 2 years of measurements.

The diurnal variations of CH_4_ (l/h) in 2 different years are shown in Figure 3. During the 2^nd^ year, the diurnal variation indicated declining emissions between 0000 and 0800 h, with the lowest emission at 0800 h. The emissions reached a peak at ~0900 h and continued with the same magnitude up to 1600 h. The CH_4_ production at this time ranged from 24 to 27 l/h. After 1600 h, the emissions declined. During the 1^st^ year, a sudden drop in CH_4_ (l/h) was observed at 1200 h. However, the rest of the hours followed a similar pattern, with more variable emissions over time.

When the CH_4_ emissions (l/h) were aggregated into time intervals (0000 to 0600 h=night; 0601 to 1200 h=morning; 1201 to 1800 h=afternoon and 1801 to 2359 h=evening), a significant difference (data were not shown) was found over 6-h intervals (*P*=0.01) during the 2^nd^ year. However, during the 1^st^ year, the CH_4_ (l/h) emissions were not different, except for lower emissions at night (*P*=0.02).

## Discussion

The results of this study have implications for the selection of cows with low CH_4_ production for breeding purposes. CH_4_ production was quantified from 2 different years for the same cows in a commercial dairy farm that were provided a similar diet in both years. Data from the same cows measured over 2 years were used to test different aspects of the variability in CH_4_ production over time.

### Key source of variation for CH_4_ production

#### Concentration of breath samples

**Figure 4:**
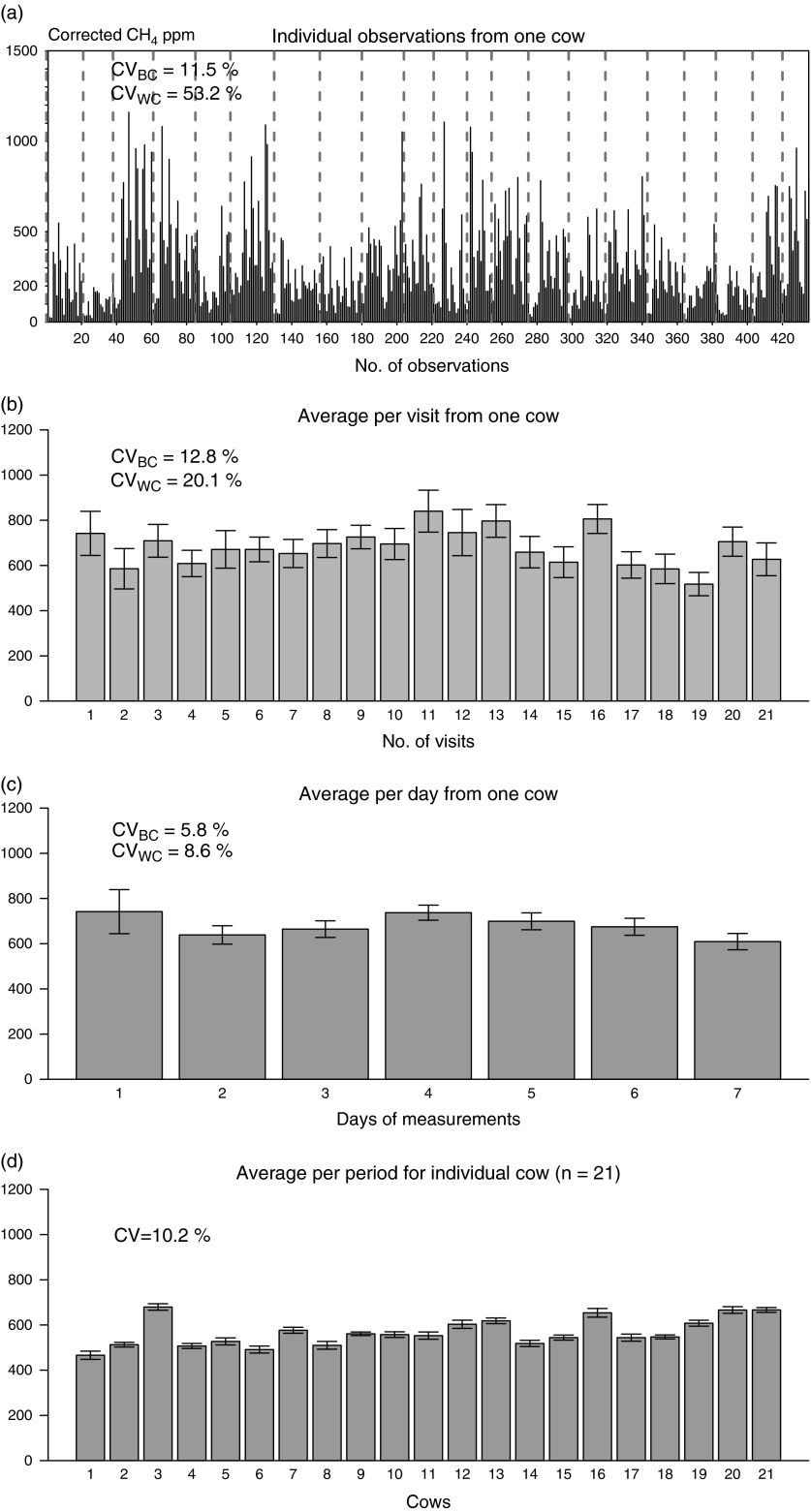
Levels of variation exist in different types of data: (a to c) are for one cow, and (d) is for 21 cows. (a) Individual observations of the concentration of corrected CH_4_ (ppm), where the broken lines separate the visits to the AMS; (b) the mean CH_4_ (l/day) (with s.e. bars) using visit-average data; and (c) the mean CH_4_ (l/day) (with s.e. bars) using day-average data. The CVs shown on (a to c) are considering 21 cows using raw data, the visit-average data and the day-average data, respectively. (d) Mean CH_4_ (l/day) (with s.e. bars) using the period average (7 days) data per cow, and the CV in this case is calculated as the s.d./expected mean. AMS=automatic milking system.

The estimation of CH_4_ production using breath samples of cows indicates considerable variation. The concentration of the breaths collected by the inlet filter of the GASMET^TM^ depends on the nose position of the cows. More importantly, the concentration of CH_4_ depends on whether the breaths and/or the eructations come from the rumen. This study showed a higher CV of the individual breath concentration (Figure 4a). The same evidence was described by Haque *et al.* (2014a) in a previous study. The substantial variation among the individual breath concentrations are a reflection of normal biological rhythms. In this connection, Garnsworthy *et al.* (2012a) stated a certain variation in eructation frequency, and the CH_4_ concentration in eructation is correlated with the differences in daily CH_4_ emissions. Unlike the respiration chamber technique, the non-invasive methods for CH_4_ estimation considered samples that had ambient exposure. Hence, some changes in the concentrations might occur. The average concentration of CO_2_ in breath typically ranges from 30 000 to 50 000 ppm. To obtain a typical breath concentration through a sampling inlet is very sporadic and is mostly influenced by the physiology of the animals and the exposure of the breath samples to the ambient air. However, trapping 2% to 3% of breath samples through the sampling device was suggested to be sufficient for a reasonably precise CH_4_ estimation from ruminants (Madsen *et al.*, 2010). In terms of variation, the individual breath concentrations show very large fluctuations that often mislead CH_4_ estimations. As shown in Figure 4, the CV gradually decreased when the visit-average (Figure 4b) or day-average (Figure 4c) data were considered. Moreover, a CV of 10.2% was found using period average data for 21 cows (Figure 4d). In this case, there is no repetition of the measurements for individual cows; hence, it is not possible to calculate within- and between-cow variations. However, these data can still be used to establish CH_4_ production with 4.5% precision (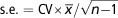, i.e., 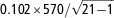
=13) for the diet when measuring for 7 days on 21 cows. To be precise in the CH_4_ estimation through breath sample analysis using the CO_2_ method, it is important to consider the mean of several individual samples, such as the emission levels per visit or per day.

#### EDMI and ECM production

Most of the studies agreed that DMI is a key factor in daily CH_4_ emission (Blaxter and Clapperton, 1965; Johnson and Johnson, 1995; Grainger *et al.*, 2007); a second key factor is determined by the digestibility of the diet (Blaxter and Clapperton, 1965; Johnson and Johnson, 1995) and the amount of concentrate or lipid supplement (Beauchemin, 2009). In this study, the EDMI and ECM had a significant influence on CH_4_ yield during both years. The effect was most likely because the increased amount of EDMI was mediated by the increased body mass and ECM production. Therefore, in a commercial farming situation, where recording individual DMI is rare, the ECM can be used to explain the variation of CH_4_ production. Higher ECM production and EDMI (kg/day) in the 2^nd^ year resulted in significantly (*P*<0.05) higher CH_4_ (l/day). The CH_4_ (l/kg EDMI) was similar in both years, which supports the fact that more CH_4_ is produced at a higher EDMI. In this connection, Boadi and Wittenberg (2002) also mentioned that 64% of the variation in CH_4_ production is explained by the DMI. The results of this study are also in line with several recent findings where diet effects on CH_4_ emissions were investigated (Beauchemin, 2009; Doreau *et al.*, 2011). In addition, Grainger *et al.* (2007) and Garnsworthy *et al.* (2012b) described similar results where DMI was mentioned as the primary determinant of CH_4_ production. Moreover, the negative correlation between CH_4_ (l/kg ECM) and the amount of ECM (kg/day) in this study revealed a reduced amount of CH_4_ per unit of product in the same line as the results previously described by Tamminga *et al.* (2007).

#### Levels of variation

In a typical feed evaluation study using a respiration chamber, the animal variation of CH_4_ production is minimized by a fixed amount of feed provided to the animals. Nevertheless, significant variation among the animals remained. A large scale CH_4_ measurement study with 215 dairy cows (Garnsworthy *et al.*, 2012b) indicated a between-cow variation of 23% (CV), whereas the within-cow variation was 6%. Based on the same data and using a mixed model, the reported variance components were 18.9% between cows and 11.5% within cows. Individual animal variations of 26.6% and 25.3% have been reported for dairy and beef heifers with *ad libitum* and restricted feeding, respectively (Boadi and Wittenberg, 2002). Blaxter and Clapperton (1965) analysed the results of 23 investigations in which sheep were offered the same amount of the same diet in contrast with another 30 investigations in which the intake was scaled according to the BW. In both analyses, the reported CV in CH_4_ emission were 7% to 8% between animals and 5% to 7% within animals. The results from 16 calorimetric studies in dairy cows with *ad libitum* feeding showed a wider range of CV (3% to 34%) in CH_4_ production (Ellis *et al.*, 2010). This large variation in CH_4_ emission was due to the wide range of DMI. Using a respiration chamber and SF_6_ tracer technique to measure CH_4_ production from lactating dairy cows that were fed *ad libitum*, Grainger *et al.* (2007) reported within- and between-cow variations of 6.1% and 19.6% for SF_6_ techniques and of 4.3% and 17.8% for the chamber techniques, respectively. Furthermore, in a study using the SF_6_ technique with four non-lactating dairy cows, Vlaming *et al.* (2008) indicated within- and between-cow variations of 6.91% to 10.09% and 6.23% to 27.79% in two diets, respectively. A wide range of individual cow variations of CH_4_ emissions (22% to 67%) were reported in a recent study with 1964 cows from 21 commercial farms (Bell *et al.*, 2014).

In the current study, the observed variation in CH_4_ (l/day) emissions between cows (5.9% to 8.8%) during 2 years is lower than those reported earlier. The range of within-cow variation (8.6% to 15.5%) over 2 years is considerably wider than the values reported by Grainger *et al.* (2007) and Garnsworthy *et al.* (2012b). However, the within-cow variation in the 2^nd^ year is in the same magnitude as mentioned by Vlaming *et al.* (2008).

Compared to the standard respiration chamber (Blaxter and Clapperton, 1965), the current study resulted in similar levels of between-cow variations and higher levels of within-cow variations. The slightly wider range of within-cow variations that were reported in this study might be linked to the greater range of EDMI and ECM production, which are assumed to be the key determinants of CH_4_ production. However, it is also related with the breath sampling length and frequency. In the present analysis only 1 day averages are used to calculate the variances, whereas a previous study showed that 5 days measurements in the AMS are needed to generate a precise CH_4_ estimation from individual dairy cows (Haque *et al.*, 2014a). Moreover, continuous measurements resulting from 8 h of placing sheep in individual pens revealed a reliable CH_4_ estimation (Haque *et al.*, 2014b). To achieve the precise variation in CH_4_ production, further study is needed to assess whether the breath sampling length and frequency is enough.

#### Repeatability and correlation of CH_4_ production over 2 years

Repeatability expresses the total variation that is reproducible among repeated measures of the same subject (Nakagawa and Schielzeth, 2010). In this study, the repeatability of CH_4_ (l/day) emissions was 0.36 and 0.41 during the 1^st^ and 2^nd^ years, respectively. The repeatability of CH_4_ emissions in the 1^st^ year was slightly lower presumably because of the higher within-cow variation. This result is similar to earlier findings in dairy cows and sheep (Vlaming *et al.*, 2008; Pinares-Patiño *et al.*, 2013). In agreement with the present study, the repeatability of the CH_4_ : CO_2_ ratio in Holstein cows was 0.37 (Lassen *et al.*, 2012), which is considered to be an effective measure for the estimation of CH_4_ production. Contrary to the present study, Pinares-Patiño *et al.* (2011) reported very low repeatability (0.16) in sheep where CH_4_ was measured using a chamber technique to rank the animals according to their emission rate.

A substantial variation in CH_4_ (l/day) emissions was observed among individual cows during the 2 years. This variation was most likely caused by the differences in the EDMI and ECM between the 2 years. However, with the adjusted ECM production (30 kg/day), the CH_4_ emissions were strongly correlated between the years. This correlation of CH_4_ (l/day) is probably related to genetic variation, that is, the heritability of CH_4_ production that was previously mentioned by Lassen *et al.* (2012) and Pinares-Patiño *et al.* (2013). The latter also stated that even after adjustment for feed intake or ECM, the trait will be repeatable. It is important to mention that cows normally show varying levels of production that ultimately results in a variable CH_4_ production. Therefore, the estimation of CH_4_ at a adjusted/standardized production is necessary in a herd, especially when ranking the cows based on CH_4_ production over different time spans. The observed correlation of CH_4_ production from individual cows in the current study could be used as an index in CH_4_ mitigation strategies by selecting low-emitter cows for the breeding process. It is worth noting that when dealing with a large number of animals for CH_4_ measurements, there will always be some individuals who are different from others because of oestrus, lameness or any other problems that affect normal feed intake, physiology, body activity or metabolism; consequently, these result in variations in CH_4_ production. Therefore, these factors should be taken into consideration.

#### Diurnal variation

A sudden drop in CH_4_ emissions (l/h) at the 1200 h during the 1^st^ year is surprising and is therefore not comparable with other reports. This is most likely the result of a fewer number of cows that visited the AMS at that specific hour, consequently producing a lower number of observations. However, the diurnal pattern of CH_4_ (l/h) in the 2^nd^ year showed identical results to the results described by Garnsworthy *et al.* (2012b). Some other methods for CH_4_ estimation, such as polytunnels grazing animals (Lockyer, 1997) and point source dispersion in grazing animals (McGinn *et al.*, 2011), showed a comparable diurnal pattern. The diurnal variation is most likely linked with the animal’s behaviour, digestive physiology and ambient condition (Garnsworthy *et al.*, 2012b), especially feeding behaviour. In the current study, feed was always available to the cows, the daily feed allocation was distributed at ~0700 h, and at ~1500 h, the remaining feed residuals were mixed and moved towards the cow. This might lead to synchronized feeding behaviour at a specific time. However, the milking time was widely different for every cow in the AMS, where milking was performed throughout a 24-h period. Therefore, the diurnal pattern might be more related to the feeding time rather than the milking time. The influence of the milking time could be considered for other methods where milking is performed, for example, twice a day at a fixed time.

## Conclusions

On a herd average basis, daily CH_4_ production was significantly higher in the 2^nd^ year as a result of a higher EDMI (kg/day). The CH_4_ emission per kg EDMI was similar throughout the 2 years. The study indicates that the key factor of variation in CH_4_ production is EDMI; this key factor can also be described by ECM production. When measuring for a short period of time, for example, a visit in the AMS or in a single day, the variation in CH_4_ (l/day) emission between cows was lower than within cows. The diurnal pattern of CH_4_ (l/h) production was influenced by the feeding behaviour of the cows and was lowest from 0000 to 0800 h. The CH_4_ production (l/day) was 51% repeatable over the 2 years. Individual cow variations over an average of 7 days show a strong positive correlation, especially when CH_4_ production is standardized using ECM in both years. This relation of CH_4_ from individual cows between the 2 years shows a potential opportunity for the selection of low CH_4_ emitter cows.
